# A Critical Review on microRNAs as Prognostic Biomarkers in Laryngeal Carcinoma

**DOI:** 10.3390/ijms252413468

**Published:** 2024-12-16

**Authors:** Kristina S. Komitova, Lyuben D. Dimitrov, Gergana S. Stancheva, Silva G. Kyurkchiyan, Veronika Petkova, Stoyan I. Dimitrov, Silviya P. Skelina, Radka P. Kaneva, Todor M. Popov

**Affiliations:** 1 Department of ENT, Medical University, 1000 Sofia, Bulgaria; 2 Molecular Medicine Center, Medical University, 1000 Sofia, Bulgaria; 3 Department of Neurology, Medical University, 1000 Sofia, Bulgaria

**Keywords:** microRNA, laryngeal carcinoma, prognosis, biomarkers, survival

## Abstract

During the past decade, a vast number of studies were dedicated to unravelling the obscurities of non-coding RNAs in all fields of the medical sciences. A great amount of data has been accumulated, and consequently a natural need for organization and classification in all subfields arises. The aim of this review is to summarize all reports on microRNAs that were delineated as prognostic biomarkers in laryngeal carcinoma. Additionally, we attempt to allocate and organize these molecules according to their association with key pathways and oncogenes affected in laryngeal carcinoma. Finally, we critically analyze the common shortcomings and biases of the methodologies in some of the published papers in this area of research. A literature search was performed using the PubMed and MEDLINE databases with the keywords “laryngeal carcinoma” OR “laryngeal cancer” AND “microRNA” OR “miRNA” AND “prognostic marker” OR “prognosis”. Only research articles written in English were included, without any specific restrictions on study type. We have found 43 articles that report 39 microRNAs with prognostic value associated with laryngeal carcinoma, and all of them are summarized along with the major characteristics and methodology of the respective studies. A second layer of the review is structural analysis of the outlined microRNAs and their association with oncogenes and pathways connected with the cell cycle (*p53*, *CCND1*, *CDKN2A/p16*, *E2F1*), *RTK/RAS/PI3K* cascades (*EGFR*, *PI3K*, *PTEN*), cell differentiation (*NOTCH*, *p63*, *FAT1*), and cell death (*FADD*, *TRAF3*). Finally, we critically review common shortcomings in the methodology of the papers and their possible effect on their results.

## 1. Introduction

During the past decade, a vast number of studies were dedicated to unravelling the obscurities of non-coding RNAs in all fields of the medical sciences [1,2]. A great amount of data has been accumulated, and consequently a natural need for organization and classification in all subfields arises. The aim of this review is to summarize all reports on microRNAs that were delineated as prognostic biomarkers in laryngeal carcinoma. Additionally, we attempt to allocate and organize these molecules according to their association with key pathways and oncogenes affected in laryngeal carcinoma. Finally, we critically analyze the common shortcomings and biases of the methodologies in some of the published papers in this area of research.

MicroRNAs (miRNAs) are a class of small (around 22 nucleotides long), non-coding, single-stranded RNA molecules [3]. MiRNAs play a role in regulating gene expression of target mRNAs post-transcriptionally. They usually act as inhibitors by degrading or repressing the translation of targeted gene transcripts, which is achieved by guiding a multi-protein silencing complex to partially complementary sites on the mRNA of target genes, therefore leading to gene silencing [4,5]. However, in some cases miRNAs can also bind to target sequences in promoters and thus act as gene activators [6]. Due to the partial complementarity of the targeted sites on the mRNA, a single miRNA can take part in the regulation of many different mRNAs, but at the same time, different miRNAs can modulate an individual mRNA [4].

Since they can recognize a variety of mRNA transcripts, through their regulatory function miRNAs can interfere with many fundamental cellular processes, including division, proliferation, differentiation, development, apoptosis, angiogenesis, etc. Abnormal expressions of some miRNAs have been shown to be involved in different pathogenic processes, including carcinogenesis [6].

With the caveat that the size and direction of the regulatory effects of some miRNAs may vary and may depend on how they interact with the effects of other miRNAs, we could simplify and classify miRNAs into two main groups, depending on how they affect the development of tumors:

- Tumor suppressor miRNAs, which negatively regulate the expression of oncogenes or genes that induce apoptosis. When downregulated, their suppressive function is decreased.
- Oncogenic miRNAs, which activate oncogenes or silence tumor suppressor genes. Their overexpression facilitates oncogenesis [5].

Recently, extensive research has been carried out on the connection between miRNAs and different types of carcinomas, including LSCC (laryngeal squamous cell carcinoma). While dysregulated expression of miRNAs in LSCC tissues and patient blood as compared with normal adjacent tissues has been observed on many occasions and has been associated with diagnostic features, more and more studies have focused on the potential prognostic value of miRNAs’ expression changes, suggesting the usefulness of miRNAs as prognostic biomarkers for that specific type of cancer.

## 2. Methods

A literature search was performed using the PubMed and MEDLINE databases with the keywords “laryngeal carcinoma” OR “laryngeal cancer” AND “miRNA” OR “miRNA” AND “prognostic marker” OR “prognosis”. Only research articles written in English were included, without any specific restrictions on study type. Two authors independently screened the data sources, and data analysis was also carried out by both authors. The search strategy is outlined in Table 1.

## 3. MiRNAs as Prognostic Biomarkers in Laryngeal Carcinoma

We have found 43 articles that report 39 miRNAs with prognostic value associated with laryngeal carcinoma, all of which are summarized in Table 2 along with the major characteristics and methodology of the respective studies.

### 3.1. MiR-19a (Cluster 17-92), Chromosome 13

The miR-17/92 cluster (also known as ‘oncomiR-1’) is one of the best-studied miR clusters and is very often dysregulated in hematopoietic and solid cancers. It is located at chromosome 13. MiR- 19a is part of that cluster along with miRNA 17-5p, miRNA 18a, miRNA 20a, miRNA 19b-1, and miRNA 92a-1. Tian-Yi Wu et al. used quantitative real-time PCR to detect miR-19a expression in fresh frozen LSCC tissues of 83 patients with LSCC stage T1–T4 [8]. Patients with high miR-19a expression levels were shown to have significantly shorter overall survival than those with low miR-19a expression levels. Using in vitro functional studies and animal xenograft tumors, Tian-Yi Wu et al. assessed the effect of miR-19a on proliferation, apoptosis, and tumor growth. After a multivariate Cox analysis, they suggested miR-19a as an independent prognostic factor of overall survival rate of LSCC patients.

### 3.2. X-Chromosome-Located MiRs: miR-20b (Cluster 106a-363) and miR-766-5p

MiR-20b is a part of the tumor-related miR-106a/363 cluster, located at the X-chromosome. T.L. Pantazis et al. studied the potential prognostic value of miR-20b in a group of 105 patients diagnosed with T1–T4-stage LSCC using real-time qPCR methodology [9]. Kaplan–Meier survival analysis showed that negative miR-20b-5p expression status predicts favorable prognosis regarding DFS (disease-free survival) and OS (overall survival) of LSCC patients. Through N-stage stratification of the patient groups, it is demonstrated that the prognostic value of miR-20b-5p holds for node-negative patients, implying its possible independence from the extent to which the cancer has spread.

Another X-chromosome-located MiR is miR-766-5p. Fu Chen et al. examined miR-766-5p as a part of the circSHKBP1/miR-766-5p/HMGA2 axis in a cohort of 60 LSCC patients (stages I–IV) [48]. The results reveal that the miR-766-5p expression level is inversely proportional to circSHKBP1 expression, implying that circSHKBP1 serves as sponge for miR-766-5p. Furthermore, overall survival was found to be shorter in LSCC patients with decreased miR-766-5p expression levels.

### 3.3. MiR-21—The Classic miR Oncogene

MiR-21 has been reported to be overexpressed and also act as oncogenic miRNA in many human malignancies. Three studies reviewed the expression of miR-21 in patients with LSCC and assessed its association with patient survival. An Hu et al. (2014) evaluated miR-21 levels by quantitative real-time PCR in 46 fresh frozen samples of patients with laryngeal carcinoma stage T1–T4 [10]. It was demonstrated that the miR-21/miR-375 ratio is extremely sensitive and specific for disease prediction, and in addition, high miR-21 expression was linked to bad prognosis. Additionally, An Hu et al. (2015) demonstrated the potential of the MiR-21/miR-375 ratio as a prognostic factor in LSCC patients [11]. The relationship between MiR-21 and survival rates was also confirmed by S. Gao et al., who arrived at that conclusion after performing MiR array analysis as well as qRT-PCR in 236 serum samples stored at −80 °C, before RNA isolation [12].

### 3.4. MiR-23a, Cluster 23a-24-2, Chromosome 19, and miRs-26a/26b

MiR-23a is a component of the miR-23a/24-2/27a cluster. X.W. Zhang et al. described the role of miR-23a in tumor progression, proving its ability to promote cell migration and invasion in laryngeal carcinoma cell cultures when overexpressed [13]. Moreover, the association of increased miR-23a levels with worse five-year survival rates was evaluated by qRT-PCR in 52 pairs of LSCC and adjacent normal tissue and subsequent statistical analysis.

The same chromosome (chromosome 19) also carries miRs-26a/26b. According to D. Liu et al., miR-26a is upregulated and positively related to poor overall survival in human LSCC [14]. In addition to the expression of miR-26a in 56 fresh frozen samples, the authors also investigated the effects of sevoflurane in cell proliferation and apoptosis using cell lines and in vivo experiments. They came to the conclusion that sevoflurane induces apoptosis and affects EMT in laryngeal carcinoma via miR-26a targeting.

MiR-26b, similarly to miR-26a, is also reported to be highly expressed and OS- and DFS-related in LSCC tissues. S. Wang et al. aimed to elucidate the mechanisms underlying miR-26b promotion of cancer proliferation [15]. They found that miR-26b knockdown induced autophagy by targeting ULK2 and inactivated the PTEN/AKT pathway in Hep-2 cells, leading to inhibition of LSCC progression.

### 3.5. MiR-31 and miR-34

The expression of miR-31 was studied by H. Qiang et al. [16], using fresh frozen pairs from a heterogeneous cohort of patients, including predominantly laryngeal and hypopharyngeal SCC, and different cell lines served as material for the analysis. A significant association of low miR-31 levels with longer survival was discovered, along with a close correlation between high miR-31 and some clinicopathological features such as poor tumor differentiation, presence of metastasis, and advanced staging.

MiR-34s form a family of three members—miR-34a, miR-34b, and miR-34c—encoded on two different chromosomes (Chr1 and Chr11). They are often regarded as tumor suppressor miRs, and we found three studies proving their downregulation in laryngeal cancer. Massimo Re et al. (2015) detected the levels of miR-34c-5p in 90 LSCC FFPE blocks by reverse-transcription qPCR in a homogeneous group of T3–T4 LSCC patients and related lower levels to unfavorable DFS, OS, and high risk of recurrence [17]. The author and his team carried out further research on this relationship (Massimo Re et al. (2017)), which confirmed their initial results [18]. Z. Shen et al. (2012) described the ability of miR-34a to suppress cell proliferation by arresting cells at the G0/G1 phase, to target survival and last but not least to alter patient outcome [19].

Apart from the effects of quantitative changes, epigenetic changes in miR-34a and their outcomes have also been analyzed. Z. Shen et al. (2016) applied bisulfite pyrosequencing technology to measure the methylation levels of the miR-34a promoter [20]. Using Kaplan–Meier survival curves, they illustrated the correlation between promoter hypermethylation and decreased overall survival.

It should be noted, however, that miR-34s cannot be unanimously classified as tumor suppressor miRNAs. Conversely to the results outlined above, I. Piotrowski et al. observed upregulation of miR-34a-5p, miR-34b-5p, and miR-34c-5p in LSCC and reported that high expression of miR-34a-5p (HR = 3.466) and miR-34b-5p (HR = 6.761) was associated with a higher hazard and lower survival rate [21].

### 3.6. MiR-93, miR-210, and miR-144b

MiR-93-5p, miR-144-3p, and miR-210-3p are all strong predictors of recurrence-free survival.

This was shown by Popov et al. (2022) through examining their expression levels in LSCC samples using microarray profiling and RT-qPCR [22]. MiR-93-5p and miR-210-3p were clearly established as oncogenes, since their upregulation was related to significantly worse survival rates. On the other hand, miR-144-3p was demonstrated to be a tumor suppressor, as it appeared to be heavily downregulated in most cases.

This is consistent with the findings of S. Y. Zhang et al., who investigated the expression of miR-144-3p in laryngeal carcinoma and its in vitro and in vivo effects and found it to be a tumor suppressor which inhibits cellular migration, invasion, EMT, and cell growth [35]. S. Y. Zhang et al. also analyzed the expression of ETS-1, which miR-144-3p suppresses, and found that lower ETS-1 expression was linked to significantly higher survival rates.

Notably, Popov et al. discovered a paradoxical relationship between survival rates and the expression of miR-144-3p in that, despite its classification as a tumor suppressor, patients with relatively high expression levels had significantly worse survival rates when compared to patients with a more pronounced downregulation. How the expression levels of those three miRs related to each other was evaluated by Popov et al. by running a Spearman’s rank-order correlation, which revealed a very strong statistically significant positive correlation between miR-93-5p and miR-210-3p, as well as a strong association between miR-93-5p and miR-144-3p and between miR-144-3p and miR-210-3.

### 3.7. Chromosome-1-Located miRs: miR-9, miR-29c, miR-101, miR-200a, and miR-137

MiR-9 is located in Chr1 and has been reported to be overexpressed in different types of cancer. S. Wu et al. focused their study on the dysregulation of miR-9 expression and its potential use as a prognostic factor of outcomes in patients with laryngeal carcinoma [7]. Levels were detected to be significantly higher in tumor tissues from 103 patients diagnosed with LSCC compared with adjacent non-cancerous tissues. Overexpression was further linked to shorter OS. MiR-9 upregulation was found to be strongly correlated with some clinicopathological parameters such as tumor differentiation, lymph node metastasis, clinical stage, and thyroid cartilage invasion.

MiR-29c can be found in the same chromosome; however, its levels of expression in LSCC (unlike miR-9) are reported to be low. R. Fang et al. extracted miR-29c from formalin-fixed, FFPE tissue samples of 66 patients by quantitative real-time PCR and associated its decreased expression with worse overall survival, along with smoking index, tumor size, tumor site, tumor differentiation, T classification, TNM stage, and lymph node metastasis [44].

MiR-101 encoding genes are located at two different chromosomes (Chr1p31.3 and Chr9p24.1). M.H. Li et al. aimed to discover the role of miR-101 in LSCC cell proliferation, invasion, apoptosis, and cell cycle regulation and applied both in vitro and in vivo methods [31]. They determined miR-101 to be a downregulated tumor suppressor miR, correlated with advanced T stage, lymph node metastasis, and poor outcome. Furthermore, they identified CDK8 as its target gene, with miR-101 functioning as a CDK8, beta-catenin, and cyclin D1 suppressor.

The miR-200 family is composed of five members (miR-200a, miR-200b, miR-200c, miR-141, and miR-429). R. Cappellesso et al. studied the role of Slug and E-cadherin, which are distinctive markers of epithelial–mesenchymal transition, in LSCC patient outcome [45]. Additionally, using a quantitative reverse-transcription PCR assay to analyze the expression of miRs extracted from FFPE block samples, they assessed their potential to alter Slug gene regulation. Moreover, the analysis revealed that in cases with disease recurrence, the expression of miR-200a and miR200c is significantly lower.

MiR-137 is also placed on chromosome 1 (1p21.3). It has been described as taking part in cell cycle regulation via the G1/S-phase checkpoint. Unlike other authors, S. Langevin et al. explored not only the quantitative changes in miR levels, but also the methylation status of miR-137 promoter by methylation-specific polymerase chain reaction [46]. The samples used in this study are from patients with HNSCC; however, it is expressly clarified that results could be applied to all different sites (oral cavity, pharynx, and larynx) with no significant differences.

### 3.8. Chromosome-2-Located miRs: miR-1246, miR-149, and miR-375

The genes of miR-1246, miR-149, and miR-375 are encoded in chromosome 2.

Q. Huang et al. investigated miR-1246 levels in both laryngeal carcinoma tissues and patients’ blood, and expression was reported to be higher in cancerous versus control samples [27]. The study pointed out small extracellular vesicles (sEV) as the prevailing form in which extracellular miR-1246 is found in samples, rather than in its soluble form. In vitro analysis showed that lowering the levels of miR-1246 in sEV form leads to inhibition of tumorigenesis and cancer progression. The mechanism of action of sEV miR-1246 was related to CCNG2 targeting. A Kaplan–Meier curve was presented, suggesting miR-1246 upregulation as a potential predictor of unfavorable prognosis.

MiR-149 and miR-375 are both reported to be downregulated and associated with adverse outcome in LSCC patients. The data presented by Y. Xu et al. indicated correlation between decreased miR-149 levels and some clinicopathological factors, such as stage, differentiation, and presence of metastasis [37]. In vitro analysis in Hep-2 cell cultures shone light on the suppressive effects of ectopic miR-149 expression on cell proliferation. Correspondingly, An Hu et al. used formalin-fixed paraffin-embedded tissue samples from 46 LSCC patients in order to assess the levels of miR-375 and suggested an association of miR-375 depletion with supraglottic tumor site and alcohol consumption [10]. Interestingly, they look at miR-375 as a part of the combined miR-21/miR-375 ratio diagnostic biomarker, which was shown to be increased in LSCC. Another team, F. Dai et al., confirmed the correlation between miR-375 and CST1, one of its putative target genes, and used LSCC cell lines to explore the suppressive effects of miR-375 on cell viability and migration and its ability to promote apoptosis [42]. Being classified as a tumor-suppressive microRNA with strong association between its dysregulation and oncogenesis, several studies were conducted to examine the potential of miR-375 as a diagnostic and prognostic biomarker in different subsites of head and neck tumors. A significant role of miR-375 downregulation was shown in detection and long-term follow-up not only in LSCC, but also in oral carcinoma [51,52].

### 3.9. Chromosome-5-Located miRs: miR-146a, miR-143, miR-145, and miR-449a

Two research teams found miR-143 to be downregulated and related to poor overall survival in patients suffering from LSCC [33,34]. Both teams used series of in vitro and in vivo analyses to examine the impact of miR-143 expression on proliferation, invasion, and migration of LSCC cells and tumor growth. According to one of the teams, L. Han et al., miR-143-3p can be described as one of the MAGE-A9-related miRNAs, revealing an inverted correlation between miR and MAGE-9 protein levels [33]. The other team, F. Zhang et al., found that miR-143 directly targeted the k-Ras gene, suppressing tumor progression via the k Ras/Raf/MEK/ERK axis [34].

Increased levels of miR-146a and miR-449a were detected in HPV-negative fresh frozen LSCC samples by I. Piotrowski et al. [21]. Based on statistical analysis, the author and his team concluded that patients with high miR-146a expression levels had poorer outcomes. Surprisingly, low miR-449a levels predicted shorter survival. In LSCC, the combined expression of miR-449a-5p, miR-6510-3p, and miR-133a-5p showed good potential as a biomarker for tumor tissues, simultaneously boasting high sensitivity and high specificity.

Another miR that is derived from the same chromosome and can be classified as prognostic for patients with LSCC is miR-145-5p. W. Gao et al. assessed both the quantitative irregularities of miR-145-5p levels in cancerous tissues and the occurring epigenetic changes, such as promoter hypermethylation, leading to the aforementioned aberrant expression [36]. Further analysis indicated an inverse relationship between miR-145-5p and FSCN1 and pointed out the combined low miR-145-5p/high FSCN1 expression as an independent marker of poor patient survival.

### 3.10. Chromosome-9-Located miRs: miR-126 and miR-181a

MiR-126 levels were measured in LSCC plasma samples by X. Sun et al., who reported a significant association between miR-126 downregulation and low grade of tumor differentiation as well as unfavorable outcome [50]. Moreover, analysis showed an inverse relationship between miR-126 and Camsap1. Therefore, high Camsap1 expression leading to activation of the processes of formation and aggregation of microtubules was found to be inhibited by miR-126.

X. Zhao et al. described the role of miR-181a in laryngeal carcinoma progression as a part of the miR-181a/GATA6 axis [47]. Advanced T stage, positive cervical lymph node metastasis, and shorter overall survival were related to low miR-181a expression in LSCC tissue samples. Additional Hep-2 cell culture analyses helped the research team to evaluate the effect of miR-181a and GATA6 levels on cell proliferation, migration, invasion, and apoptosis. MiR-181a/SIRT1 is another axis known to be dysregulated in tumors of different types and sites [53]. X.M. Yu et al. used qRT-PCR and immunohistochemical methods to assess the levels of SIRT1 in samples from patients with laryngeal and hypopharyngeal carcinoma and found a statistically significant correlation between low SIRT1 levels and advanced clinical stage as well as positive lymph node metastasis [54].

### 3.11. Chromosome-14-Located miRs: miR-203 and miR-300

The downregulation of miR-203 was determined by L. Tian et al. to go hand in hand with a low grade of differentiation, advanced stages, positive lymph nodes, and, last but not least, poor five-year survival rate in patients with laryngeal squamous cell carcinoma [40]. A combination of in vitro and in vivo experiments revealed the ability of miR-203 to suppress LSCC cell proliferation, invasion, and tumor growth and more importantly to target the ASAP1 gene, to promote the E-cadherin expression, and to lower the CD44 (marker of cancer stem cells) levels.

Unlike miR-203, miR-300, located in the same chromosome, was reported by F.Y. He et al. to be notably upregulated in LSCC tissues and altered according to cervical lymph node status and TNM stage [41]. A Kaplan–Meier curve was used to illustrate that lower levels of miR-300 were linked to decreased OS.

### 3.12. Chromosome-17-Located miRs: miR-301a, miR-632, miR-10a, and miR-195

MiR-301a and miR-632, both located at chromosome 17, were observed to be significantly upregulated in laryngeal carcinoma samples. Y. Lu et al. conducted in vitro analysis showing that miR-301a knockdown resulted in suppressed proliferation, duplication, and colony formation along with activation of apoptosis, G0/G1 cell cycle arrest, and alteration of some of the most representative EMT markers: E-cadherin, N-cadherin, vimentin, MMP2, and MMP [25]. Y. Lu and their team identified Smad4 as a miR-301a target and outlined the combined high miR-301a-3p/low Smad4 status as an independent prognostic marker of adverse outcome. In contrast, Y.C. Cao et al. used blood samples to assess the correlation between increased miR-632 levels and low differentiation, positive lymph nodes, advanced stage, and poor OS and DFS [30].

Opposingly, miR-10a expression was found to be decreased in serum samples from patients suffering from LSCC. S. Gao et al. used an miR array method to detect miR-10a levels and further statistical analysis to explore their association with different clinicopathological factors and five-year survival rate [12]. The team examined miR-10a as a part of the combined miR-21/miR-10a marker and concluded that a higher ratio correlated to worse survival.

In two mutually complementary articles, both published in 2017, Y. Shuang et al. laid out their findings of a significant downregulation of miR-195 in LSCC tissues and linked its lowered expression to late T stage, positive lymph nodes, advanced clinical stage, distant metastasis, and poor prognosis [38,39]. MiR-195 possibly restrained growth, invasion, migration, and colony formation of tumor cells, induced G1 cell cycle arrest, and promoted apoptosis through targeting and suppressing DCUN1D1 and Bcl-2. A high miR-195/low DCUN1D1 expression combination was shown to imply longer overall survival and vice versa.

### 3.13. Chromosome-19-Located miRs: miR-196b and miR-519a

MiR-196b can be classified as oncogenic miRNA. Two studies reported its upregulation in LSCC tissues and suggested its potential as prognostic marker of poor outcome. M. Luo et al. described a statistically significant relationship between miR-196b levels and tumor stage and differentiation [23]. Furthermore, they identified PCDH-17 as a direct miR-196b target. On the other hand, X. Zhao et al. found that miR-196b and SOCS2 expressions were inversely correlated, and through that relationship, miR-196b boosted proliferation and invasion and suppressed apoptosis in LSCC cell lines [24].

The possible use of miR-519a, located at the same chromosome, as a marker of prognosis in laryngeal carcinoma was studied by Z. Shen et al. [43]. They used the reverse-transcription PCR method in 96 LSCC samples and observed that decreased miR-519a levels were correlated to worse disease-free survival. Moreover, LSCC cell cultures were used to observe the negative effects of miR-519a on cell growth and its ability to cause G2/M cell cycle arrest. HuR was found to be a miR-519a target gene. However, protein levels were regulated without an impact on mRNA levels.

### 3.14. MiR-1205 Chr8, miR-378c-5p Chr10, miR-125b-5p Chr11, miR-296-5p Chr20, and miR-155 Chr 21

MiR-1205 dysregulation and prognostic significance in laryngeal cancer was explored by P. Li et al. [49]. They reported a downregulation of miR-1205 in patients with LSCC and its association with clinicopathological markers, such as clinical stage, T stage, and lymph node metastasis. Remarkably, this article determined a strong association between miR-1205 and E2F1, an oncogene playing a pivotal role in laryngeal carcinogenesis. E2F1 was not only found to be a target of miR-1205, but also an inhibitor of its expression, through binding to its promotor.

In order to assess the expression of miR-378c-5p in laryngeal carcinoma, Y. Xu et al. used samples from patients stratified in three groups—patients with LSCC, patients with vocal cord polyposis, and healthy controls [26]. High levels of miR-378 were related to the presence of LSCC, low grade of differentiation, and advanced stage. Postoperative control samples were taken, which allowed the authors to observe miRNA-378 expression over time. A significant decline in expression was detected in both LSCC and VCP groups six months after the surgery, with miRNA-378 levels being significantly higher in patients with tumor recurrence. High miR-378c-5p expression was also linked to higher hazard and lower survival rate in laryngeal cancer by I. Piotrwski et al. [21].

F. Chen et al. published an article designating miR-125-5p as a tumor suppressor miR which leads to poor overall survival in LSCC patients when downregulated [32]. Cell cultures and animal xenograft models were used to determine the role of miR-125-5p as a cell growth and glycolysis inhibitor, a promoter of apoptosis and, last but not least, as a regulator of MAP3K9 levels.

The expression of miR-296-5p was studied in a small homogenous cohort of 34 patients suffering from early-stage LSCC, treated with radiotherapy only. D. Maia et al. observed a statistically significant correlation between increased miR-296 levels and both radioresistance and recurrence of the disease [28].

MiR-155, encoded in chromosome 21, was analyzed and reported to be higher in laryngeal carcinoma patients by X. Zhao et al. [29]. The analysis showed an association between its upregulation and advanced stages of the disease, low grade of differentiation, and positive lymph node status. Moreover, it implied a positive relationship between miR-155 and both YB-1 and c-Myb genes, and a Kaplan–Meier curve was used to illustrate the statistically significant relationship between the expression of YB-1 and the unfavorable prognosis of patient survival.

### 3.15. MiRNAs in Plasma

The main focus in this review has been set to be prognostic miRNA biomarkers in tumor tissue samples (fresh frozen FFPE, etc.), but for the sake of thoroughness, we are obliged to also briefly discuss plasma samples as sources of miRNAs for prognosis. A liquid biopsy is an emerging technology which consists in sampling non-solid body tissues (usually blood and saliva) and analyzing them for different circulating biomarkers, including microRNAs. Liquid biopsies could be an effective way of diagnosing and monitoring cancer because of how non-invasive, rapid, and repeatable they are [55]. Studies have already shown liquid biopsies to have an application for detecting oral cancer, as well as cancer located in other head and neck subsites [56], with circulating microRNAs functioning as biomarkers. The main reason liquid biopsies are still not widely used for screening is the poor result accuracy of the current technology when applied in a clinical setting. Even under academic conditions, problems with reporting and inconsistencies in collecting, storing, and processing samples compromises the results of studies in that area [57]. On some occasions, analysis of different types of samples reaches congruent conclusions. For example, as we already discussed in Section 3.3, both An Hu et al. (2014) [11], using fresh frozen samples, and S. Gao et al. [12], using serum samples, independently showed a relation between MiR-21 expression and LSCC survival rates. Another case, presented in Section 3.8 above, is that of Huang Q. et al. [27], who used a combination of sample types and proved that miR-1246 is overexpressed in both plasma sEV and LSCC tissues and that such overexpression is linked to worse prognosis. However, care should be taken when comparing miRNA expression in liquid biopsies and miRNA expression in tissue biopsies, as those should not necessarily be considered identical metrics. Before hypothesizing the predictive power of miR-21 serum levels in their article, S. Gao et al. [12] explicitly stated that relevant previous studies had focused almost exclusively on tissue samples and that, as of then, the link between circulating MiR-21 levels and LSCC prognosis had remained unknown.

## 4. Systematic Classification of miRs Exhibiting Prognostic Significance Based on Their Association with Pivotal Altered Pathways and Oncogenes in Laryngeal Carcinoma

**Figure 1:**
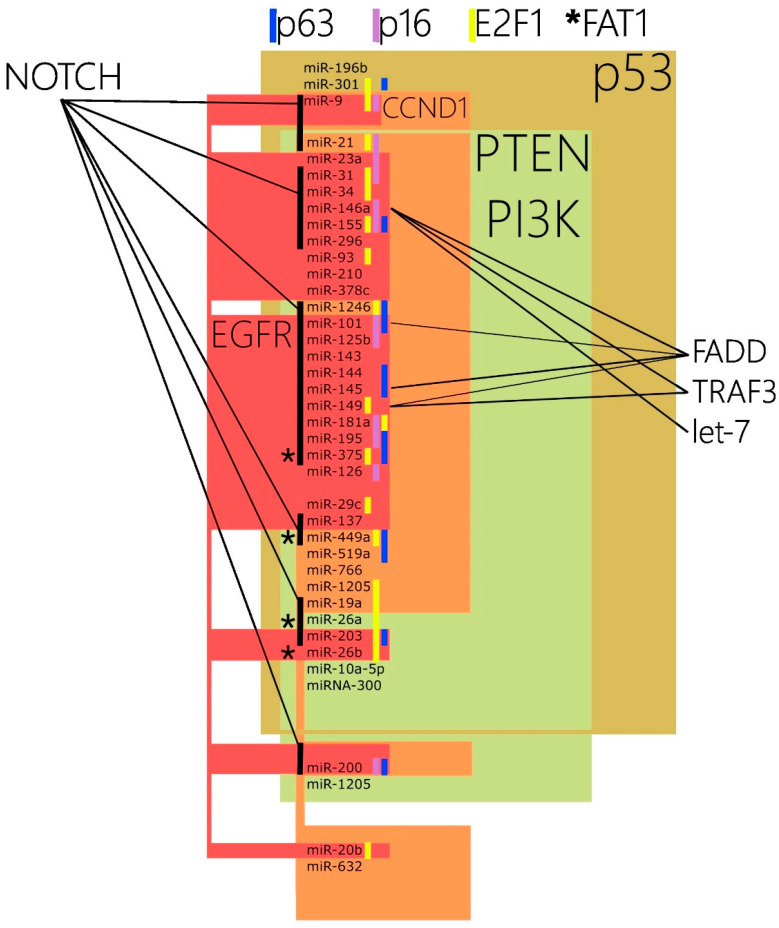
Representation of miRs with prognostic value in laryngeal carcinoma and their association with major oncogenes/pathways (Euler diagram). Asterix displays available data for association with *FAT1* gene.

We reviewed all available cancer studies that reported any molecular association between the most prominently affected oncogenes and pathways in laryngeal carcinoma [58] and the miRs that we have found to possess prognostic value in the literature. We have a summary of this deep analysis of the literature given in Table 3 along with the respective references. Additionally, we have prepared a Venn diagram to illustrate the collected data (Figure 1).

### 4.1. MiRs Demonstrating Prognostic Significance and Their Association with Oncogenes and Pathways Connected with the Cell Cycle: p53, CCND1, CDKN2A/p16, and E2F1

Out of the 43 (86%) referenced miRs with prognostic value, 37 are found to be in molecular association with p53 and its molecular pathway (Table 3). In most of the cases, we have found a sufficient number of papers for each miR reporting such a relationship. The percentage astoundingly corresponds to the percentage of p53 mutation—84%—found in patients with squamous cell carcinoma of the head and neck [58]. These findings suggest that most of the prognostic biomarkers are in regulatory co-dependency with the p53 oncogene. Only miRs miR-9, miR-20b, miR-196b, miR-301a, and miR-632 lack the data to be in a common regulatory network. Exclusively, a study from 2014 [59] reports that miR-9 is activated by HPV E6 in a p53-independent manner. We found that 79.5% (31 out of the 39 miRs) are reported to be in a regulatory dependence with the Cyclin D1 (CCND1) gene. MiR-19, miR-26a/b, miR-31, miR-196b, miR-10a, miR-203, and miR-300 are not reported in the literature to be associated with CCND1. Moreover, two studies confirm the functional 31 [111]/miR-10a [180]. Only 25.6% of the miRs that have prognostic value are reported to have common regulatory associations with CDKN2A/p16. A higher number of associated miRs is found when E2F1 (46%) is analyzed. This higher rate of association in comparison to p16 can be explained by the fact that E2F1 is involved in cell proliferation, migration, and cell cycle in both HPV-positive and HPV-negative tumors. Interestingly, we can see from Table 3 that only two miRs overlap in terms of regulatory networks for both CDKN2A/p16 and E2F1: miR-9 and miR-181a. Generally, we could sum up these findings as follows: (1) Almost all miRs with prognostic value are in regulatory association with cell cycle oncogenes (data lack only for miR-196b to be in any regulatory relationship with the cell cycle oncogenes). (2) Predominantly, biomarker miRs are associated with p53 and Cyclin D1. (3) According to available data, regulatory association of biomarker miRs with E2F1 excludes such connections with CDKN2A/p16 and vice versa—in terms of dependence, both oncogenes divide biomarker miRs in two distinct subgroups (chi-square, *p* < 0.05). Some of those differentiations are backed up with studies reporting regulatory independence—see Table 3 [82,112,144,186,239,260,283].

### 4.2. MicroRNAs Demonstrating Prognostic Significance and Their Association with Oncogenes and Pathways Connected with the RTK/RAS/PI3K Cascades: EGFR, PI3K, and PTEN

A very high percentage of the reviewed miRs—89.7% (35 out of 39)—were found to have regulatory links with the PTEN/PI3K cascade. Only miR-20b, miR-632, miR-200a, and miR-1205 lack the data to be in a common regulatory network. We have to acknowledge that the majority of dysregulated miRs with prognostic value are functionally connected in some way with the PTEN/PI3K pathway despite the fact that the percentage of genetic alterations in this cascade is within at least an order of magnitude less—ranging between 6% and 56% of the cases [58]. This is a major difference when comparing the same miRs and their relation to cell cycle oncogenes and pathways, where the percentages generally correspond. These findings would suggest that while not always genetically altered, the PTEN/PI3K cascade is phenotypically a key player for tumor progression and prognosis. Similar dissociation is evident with EGFR—only 15% of the HPV-negative patients and 6% of the HPV-positive patients have genetic alterations of EGFR [58], and a striking 64% of the dysregulated biomarker miRs from our list are found to have a regulatory effect on EGFR. These data indirectly suggest that EGFR and PI3K/PTEN pathways play a very significant role in laryngeal cancer progression and prognosis that extends far beyond the context of their genetic alterations’ frequency in these patients.

### 4.3. MiRs Demonstrating Prognostic Significance and Their Association with Oncogenes and Pathways Connected with Cell Differentiation: NOTCH, p63, and FAT1

Of the biomarker miRs, 59% were found be associated functionally with the NOTCH cascade and only 25.6% with p63. The frequency of genetic alterations in NOTCH in patients with squamous cell carcinoma revolve around 17% and 26%, whereas for p63, 19% and 28%, for HPV-negative and HPV-positive carcinoma, respectively [58]. Despite the very high rate of mutations in FAT1 (32%), we have found only four miRs from our list that are reported to have regulatory connections with this gene. We cannot assess whether the available data in the literature are just not sufficient or whether, at least from a prognostic point of view, this molecular domain does not have such a major role.

### 4.4. MiRs Demonstrating Prognostic Significance and Their Association with Oncogenes and Pathways Connected with Cell Death: FADD and TRAF3

We have looked into cell death domain by choosing FADD and TRAF3 as the most prominently genetically altered genes. In 32% of the HPV-negative head and neck cancers, FADD is found to carry driver mutations, whereas TRAF3 is genetically altered in 22% of the HPV-positive patients [58]. Despite these significant rates, we could find almost no data that could connect those two oncogenes with the list of miRs with prognostic value in laryngeal carcinoma. Only miR-146a is reported to be regulatorily associated with both FADD [136] and TRAF3 [137]. Additionally, miRs miR-101, miR-145, and miR-149 are connected functionally with FADD [197,234,242]. Similarly to FAT1, we cannot assess whether the available data in the literature are just not sufficient or whether this molecular domain does not carry such a weight from a prognostic standpoint.

## 5. Critical Remarks on Publications Reporting Prognostic Biomarkers

After a thorough analysis of the methodology of the publications that report miRs as prognostic markers in laryngeal carcinoma, we have found a number of major concerns that are present almost ubiquitously throughout the reviewed papers. The first very important shortcoming among the majority of the studies is the inclusion of a very wide range of stages in the studied cohort. From 43 articles that report miRs as prognostic biomarkers, only four have focused their research on a more homogenous group of patients [21,22,23,53], e.g., only advanced stage (stage T3/T4 or at least stage III/IV) or early-stage carcinoma. The vast majority of the papers included patients ranging across all the tumor stages (see Table 2). This is a predicament of bias in the design of the study, since it is a well-established fact that a great number of oncogenes/dysregulated molecules are consistently upregulated vs. their matching controls in a grade- and stage-dependent manner [310]. If we conscientiously take into account this significant bias, we cannot assess whether the degree of dysregulation of those miRs is not just a correlation with stage and we are measuring its stage-dependent prognostic value. From this perspective, one might conjecture that these dysregulated miRs may serve as biomarkers not solely for strict prognostic purposes, but rather as more generalized indicators of molecular tumor progression.

A critical consideration in survival analysis is the endpoint measurement, particularly in the context of Kaplan–Meier analysis. Focusing solely on overall survival metrics (69% of the reviewed papers) may obscure the significance of specific molecular signatures related to recurrence, particularly in smaller cohorts. To elucidate these molecular signatures effectively, authors should prioritize recurrence-free survival/disease-free survival as the primary endpoint. This approach will mitigate confounding factors associated with other causes of death within the studied population.

Finally, the standardization of treatment protocols within the studied cohort is a critical factor for accurately assessing the true value of a molecular signature in predicting survival outcomes. The presence of heterogeneous treatment protocols introduces additional non-molecular variables that may complicate the interpretation of results.

### Regarding Other Reviews and Meta-Analyses on the Current Topic

Emerging research and accumulation of novel data on expression changes of different microRNAs in LSCC and their potential use as diagnostic and prognostic biomarkers are setting the stage for the development of new, more advanced methods for earlier detection and more efficient treatment, which warrants further efforts to organize, classify, and study that new data. There are already some research teams who have published systematic reviews and meta-analyses on prognostic microRNA biomarkers in LSCC [311,312], as well as authors who report the interrelation between microRNAs and some of the key pathways of signalization in laryngeal carcinoma [313]. In our study, we have searched all of the currently available literature and have listed all of the microRNAs for which a significant relationship between their expression level and the survival status of patients suffering from laryngeal carcinoma has been confirmed. We have attempted to organize and graphically illustrate their association with major signaling pathways and oncogenes, altered in LSCC. Last but not least, we have outlined some of the main shortcomings in the methodologies and study design characteristics of the cited articles, which must be taken into consideration when reading, analyzing, and interpreting their results.

## 6. Conclusions

MiRNA molecules constitute a field in molecular medicine that presents vast opportunities for finding quality biomarkers in cancer research. A wide range of miRNAs have been shown to correlate with various clinicopathological parameters, including tumor stage, lymph node metastasis, and patient survival. These miRNAs can act as oncogenes or tumor suppressors, and their dysregulation significantly impacts the progression of LSCC. Despite the significant number of miRNAs that are found to correlate with survival outcome in laryngeal carcinoma, validation in clinical practice and reproducibility of results are still not evident in the current published literature. Strict homogeneity of cohorts and meticulous standardizations of surgical/treatment protocols are required to sift out real prognostic biomarkers from more general indicators of molecular tumor progression. Reproducibility of results is a major necessity in order to progress from single-study results towards clinically significant molecular subtypization.

## Figures and Tables

**Table 1:** Search strategy summary.

The Search Strategy Summary	
Items	Specification
Databases and other sources searched	PubMed/MEDLINE
Search terms used	“laryngeal carcinoma” OR “laryngeal cancer” AND “microRNA” OR “miRNA” AND “prognostic marker” OR “prognosis”
Timeframe	2002–2024
Inclusion and exclusion criteria	Inclusion criteria: without predefined restriction as to the study type Exclusion criteria: restricted to articles published in English
Selection process	K.K. and T.P. independently screened data sources. Data analysis was conducted by K.K. and T.P.

**Table 2:** Studies reporting microRNAs with prognostic value associated with laryngeal carcinoma along with methodology and patient characteristics.

Ref.	MicroRNA	Stage	Method	Sample Material	Poor Prognosis	Endpoint Measurement
[7]	miR-9	T1–T4	qRT-PCR	Fresh frozen	Upregulated	OS
[8]	miR-19a	T1–T4	qPCR	Fresh frozen	Upregulated	OS
[9]	miR-20b-5p	T1–T4	RT-qPCR	Fresh frozen	Upregulated	OS; DFS: RFS
[10,11,12]	miR-21	T1–T4	Microarray qRT-PCR	Fresh frozen Blood sample	Upregulated	OS
[13]	miR-23a	T1–T4	qRT-PCR	Fresh frozen	Upregulated	OS
[14,15]	miR-26a/b	T1–T4 I–IV	RT-qPCR	Fresh frozen Blood sample	Upregulated	OS OS; DFS
[16]	miR-31	I–IV	qRT-PCR	Fresh frozen	Upregulated	OS
[17,18,19,20,21]	miR-34	T1–T4 I–IV T3–T4 T3–T4 T1–T4	RT-qPCR Bisulfite pyrosequencing	Fresh frozen FFPE	Upregulated Downregulated Hypermethylation	OS; DFS
[22]	miR-93-5p	T3–T4	Microarray RT-qPCR	Fresh frozen	Upregulated	RFS
[21]	miR-146a-5p	T1–T4	RT-qPCR	Fresh frozen	Upregulated	OS
[23,24]	miR-196b	T1–T4 T2–T4	qRT-PCR	Fresh frozen	Upregulated	OS
[22]	miR-210-3p	T3–T4	Microarray RT-qPCR	Fresh frozen	Upregulated	RFS
[25]	miR-301a-3p	T1–T2 (6 patients) T1–T4	qRT-PCR	Fresh frozen FFPE	Upregulated	OS
[21,26]	miR-378c-5p	T1–T4 I–IV	RT-qPCR	Fresh frozen Blood sample	Upregulated	OS; RFS
[27]	miR-1246	T1–T4	qRT-PCR	Fresh frozen FFPE Blood sample	Upregulated	OS
[28]	miR-296-5p	T1–T2	TLDA qRT- PCR	FFPE	Upregulated	DFS
[29]	miR-155	T2–T4	qRT-PCR	Tissue samples (not specified)	Upregulated	OS
[30]	miR-632	I–IV	RT-PCR qRT-LSCCR assay	Serum samples	Upregulated	OS; DFS
[31]	miR-101	T1–T4	qRT-PCR	Fresh frozen FFPE	Downregulated	OS
[32]	miR-125b-5p	Not specified	qRT-PCR	Fresh frozen	Downregulated	OS
[33,34]	miR-143-3p	I–IV	Microarray RT-qPCR	Fresh frozen	Downregulated	OS
[22,35]	miR-144-3p	T3–T4	Microarray RT-qPCR	Fresh frozen FFPE	Downregulated Upregulated	OS; RFS
[36]	miR-145-5p	I–IV	qRT-PCR	Fresh frozen	Downregulated Hypermethylated	OS
[37]	miR-149	I–IV	qRT-PCR	Fresh frozen	Downregulated	OS
[38,39]	miR-195	T1–T4 I–IV	qRT-PCR	Fresh frozen	Downregulated	OS
[40]	miR-203	T1–T4	qRT-PCR	Fresh frozen	Downregulated	OS
[41]	miR-300	T1–T2	qRT-PCR	Fresh frozen	Downregulated	OS
[42]	miR-375	T1–T4	qRT-PCR	Fresh frozen	Downregulated	OS
[21]	miR-449a-5p	T1–T4	RT-qPCR	Fresh frozen	Downregulated	OS
[43]	miR-519a	I–IV	qRT-PCR	Fresh frozen	Downregulated	DFS
[44]	miR-29c-3p	T1–T4	qRT-PCR	FFPE	Downregulated	OS
[45]	miR-200a/c	T1–T4	qRT-PCR	FFPE	Downregulated	RFS
[46]	miR-137	T1–T4	Methylation-specific PCR qRT-PCR	FFPE	Hypermethylated	OS
[47]	miR-181a	T2–T4	qRT-PCR	Tissue samples (not specified)	Downregulated	OS
[48]	miR-766-5p	I–IV	qRT-PCR	Tissue samples (not specified)	Downregulated	OS
[49]	miR-1205	T1–T4	qRT-PCR	Tissue samples (not specified)	Downregulated	OS; DFS
[50]	miR-126	T1–T4	qRT-PCR	Tissue samples (not specified) Blood samples	Downregulated	OS
[12]	miR-10a-5p	T1–T4	Microarray qRT-PCR	Blood sample	Downregulated	OS

OS—Overall survival. DFS—disease-free survival. RFS—recurrence free survival. Numbers in the table represent references.

**Table 3:** Studies reporting molecular association between the most prominently affected oncogenes and pathways in laryngeal carcinoma and miRs with prognostic value. Numbers in the table represent references. Light green–existent data for regulatory association; grey–lack of regulatory association.

MicroRNA	p53	PTEN/PI3K	CCND1	CDKN2A /p16	EGFR	NOTCH	P63	FAT1	FADD	TRAF3	E2F1
miR-9	[59]	[60,61]	[62]	[63]	[64]	[65]					[66]
miR-19a	[67]	[68]				[69]					[70]
miR-20b-5p			[71]		[72]						[73]
miR-21	[74,75]	[76,77]	[78]	[79]	[80]	[81]					[82]
miR-23a	[83,84,85]	[86,87]	[88]	[89]	[90]						
miR-26a	[91,92,93]	[94,95]			[96]	[97]		[98]			[99]
miR-26b	[100,101]	[102,103]			[104]			[98]			[105]
miR-31	[106,107,108]	[109,110]	[111]	[112]	[113]	[114]					[115]
miR-34	[116,117]	[118,119]	[120]		[121]	[122]					[123]
miR-93-5p	[124]	[125,126]	[127]		[128]						[127]
miR-146a-5p	[129,130]	[131,132]	[130]	[133]	[134]	[135]			[136]	[137]	
miR-155	[138,139,140]	[141,142]	[143]	[144]	[145]	[146]	[147]				[148]
miR-196b		[149,150]									
miR-210-3p	[151,152]	[153,154]	[155]		[156]						
miR-296-5p	[157]	[158]	[159]			[160]					
miR-301a-3p		[161,162]	[163]				[164]				[165]
miR-378c-5p	[166,167]	[168]	[166]		[169]						
miR-632			[170]								
miR-1246	[171,172]	[173]	[174]			[175]	[171]				[174]
miR-10a-5p	[176,177]	[178,179]	[180]								
miR-29c-3p	[181,182]	[183,184]	[185]	[186]	[187]						[188]
miR-101	[189,190]	[191,192]	[26]	[193]	[194]	[195]	[196]		[197]		
miR-125b-5p	[198,199]	[200,201]	[202]	[203]	[204]	[205]	[206]				
miR-126	[207]	[208,209]	[210]	[211]	[212]						
miR-143-3p	[213,214]	[215,216]	[217]		[218]	[219]					
miR-144-3p	[220]	[221,222]	[223]		[224]	[225]	[226]				
miR-145-5p	[227,228]	[229,230]	[231]			[232]	[233]		[234]		
miR-149	[235,236]	[237]	[238]	[239]	[240]	[241]			[242]		[243]
miR-181a	[244,245]	[246]	[247]	[248]	[249]	[250]					[251]
miR-195	[252,253]	[254,255]	[256]	[257]	[258]	[259]					[260]
miR-200a	[261,262]		[263]	[264]	[265]	[266]	[267]				
miR-203	[268,269]	[270]			[271]	[272]	[273]				[274]
miR-300	[275,276]	[277]		[278]							
miR-375	[279,280]	[281]	[282]	[283]	[284]	[285]		[286]			[287]
miR-449a-5p	[288,289]	[290]	[291]			[292]	[267]	[293]			[294]
miR-519a	[295]	[296]	[297]				[298]				
miR-766-5p	[299]	[300]	[301]								
miR-1205	[302]		[303]								[50]
miR-137	[304,305]	[306]	[307]	[28]	[308]	[309]					

## Abbreviations

mRNA	Messenger RNA
miRNA	MicroRNA
miR	MicroRNA (often used as a prefix for specific miRNAs)
LSCC	Laryngeal Squamous Cell Carcinoma
DFS	Disease-Free Survival
OS	Overall Survival
FFPE	Formalin-Fixed Paraffin-Embedded (FFPE)
qPCR	Quantitative Polymerase Chain Reaction
HNSCC	Head and Neck Squamous Cell Carcinoma
sEV	Small Extracellular Vesicles
FADD	Fas-Associated Death Domain
EMT	Epithelial–Mesenchymal Transition
HR	Hazard Ratio
TNM	Tumor, Node, Metastasis (staging system)
p53	Tumor Protein p53 (a tumor suppressor)
PTEN	Phosphatase and Tensin Homolog
PI3K	Phosphoinositide 3-kinase
CCND1	Cyclin D1
CDKN2A	Cyclin-Dependent Kinase Inhibitor 2A
/p16	A specific protein encoded by CDKN2A, often referred to as p16INK4a
EGFR	Epidermal Growth Factor Receptor
NOTCH	A family of genes that encodes transmembrane receptors
P63	Tumor Protein p63
FAT1	FAT Tumor Suppressor Protein
TRAF3	TNF Receptor-Associated Factor 3
E2F1	E2F Transcription Factor 1
circSHKBP1	Circular RNA SHKBP1
ETS-1	E26 Transformation-Specific Sequence 1
CDK8	Cyclin-Dependent Kinase 8
CCNG2	Cyclin G2
AKT	Protein Kinase B
